# Influence of Raster Pattern on Residual Stress and Part Distortion in FDM of Semi-Crystalline Polymers: A Simulation Study

**DOI:** 10.3390/polym14132746

**Published:** 2022-07-05

**Authors:** Anto Antony Samy, Atefeh Golbang, Eileen Harkin-Jones, Edward Archer, Monali Dahale, Marion McAfee, Behzad Abdi, Alistair McIlhagger

**Affiliations:** 1 Engineering Research Institute, Ulster University, Shore Road, Newtownabbey, Co., Antrim BT37 0QB, UK; e.harkin-jones@ulster.ac.uk (E.H.-J.); e.archer@ulster.ac.uk (E.A.); m.dahale@ulster.ac.uk (M.D.); b.abdi@ulster.ac.uk (B.A.); a.mcilhagger@ulster.ac.uk (A.M.); 2 Northern Ireland Advanced Composites and Engineering (NIACE) Centre, Airport Road, Belfast BT3 9DZ, UK; 3 Centre for Mathematical Modelling and Intelligent Systems for Health and Environment (MISHE), Atlantic Technological University, ATU Sligo, Ash Lane, F91 YW50 Sligo, Ireland; mcafee.marion@itsligo.ie; 4 Centre for Precision Engineering, Materials and Manufacturing (PEM Centre), Atlantic Technological University, ATU Sligo, Ash Lane, F91 YW50 Sligo, Ireland

**Keywords:** Fused Deposition Modeling (FDM), semi-crystalline polymers, warpage, residual stress, simulation, raster pattern

## Abstract

In fused deposition modelling (FDM) based on the selected raster pattern, the developed internal thermal residual stresses can vary considerably affecting the mechanical properties and leading to distinct part distortions. This phenomenon is more pronounced in semi-crystalline than amorphous polymers due to crystallisation. Hence, this study focuses on the simulation of the FDM process of a semi-crystalline polymer (polypropylene) with raster patterns such as line (90°/90°), line (0°/90°), zigzag (45°/45°), zigzag (45°/−45°), and concentric from Cura (slicing software). The simulation provides visualisation and prediction of the internally developed thermal residual stresses and resulting warpage with printing time and temperature. The sample with a line (90°/90°) raster pattern is considered as the reference sample in order to compare the relative levels of residual stress and warpage in the other printed/simulated samples. Among the considered raster patterns, the concentric pattern displays the lowest amount of warpage (5.5% decrease) along with a significant drop in residual stress of 21%. While the sample with a zigzag (45°/−45°) pattern showed the highest increase of 37% in warpage along with a decrease of 9.8% in residual stresses. The sample with a zigzag (45°/45°) pattern, exhibited a considerable increase of 16.2% in warpage with a significant increase of 31% in residual stresses. Finally, the sample with a line (0°/90°) raster pattern displayed an increase of 24% increase in warpage with an increase of 6.6% in residual stresses.

## 1. Introduction

Additive Manufacturing (AM) also referred to as 3D printing, offers a wide range of techniques that are capable of manufacturing complicated geometrical parts efficiently using materials such as plastics, metals, and ceramics [1,2]. Among the various available AM techniques, Fused Deposition Modelling (FDM) is one of the most commonly employed AM methods due to its potential to manufacture parts with complex geometries, cost efficiency, and simplicity in operation and design [3,4,5,6,7]. Advantages of FDM include a wide range of feedstock materials including metals and thermoplastic polymers [8], and it is economic and user-friendly.

In FDM, thermoplastic polymers are predominantly employed as feedstock materials [3,8,9,10]. Compared to semi-crystalline polymers, amorphous polymers are preferred, due to their lower propensity for warpage. Semi-crystalline polymers on the other hand display significant warpage and they are challenging to print by FDM [11]. Crystallization of semi-crystalline polymers is highly temperature dependent and hence is greatly influenced by the printing parameters of FDM. Several researchers have investigated the effects of printing parameters such as the print bed temperature, ambient temperature, print speed/nozzle speed, layer thickness of the printed part, etc. [12,13,14,15,16,17,18].

A raster pattern (also referred to as infill pattern/tool path) is the orientation in which the roads are deposited while a part is being printed. The orientation of these selected patterns can directly influence the strength, weight, printing time, and accumulated stresses which can lead to the development of cracks and delamination [19,20]. Hence, the effect of raster pattern on mechanical properties has been a point of interest in the literature. Several researchers have conducted studies on various raster patterns to examine their influence on tensile strength [21,22,23], compression [20,24], and flexural [25] properties.

However, only a small number of studies have examined the effect of raster pattern on the resulting part distortion of an FDM fabricated polymeric part [26]. Simulation of the effect of raster pattern on part distortion in the FDM process has not been reported before in the literature. Compared to the other printing parameters, the raster pattern does have a considerable effect on the structural integrity of a FDM fabricated part.

Among the commonly used raster patterns in FDM, concentric pattern is reported to have low deformation and superior mechanical properties [27,28], followed by the zigzag pattern (45°/−45°) which has also been reported to produce parts with high mechanical properties [25,29]. Depending on the selected raster pattern, the heat transfer and cooling pattern of a FDM printed part can change considerably giving rise to thermal residual stresses leading to varying residual stress accumulation affecting the mechanical properties of the part. Therefore, this study aims to focus on understanding the effect of various raster patterns on the accumulated residual stresses and warpage of a FDM printed part and relating them to their mechanical properties.

In our previous works, along with line (90°/90°) raster pattern, zigzag (45°/45°) was investigated. Additionally, in this work raster patterns such as line (0°/90°), zigzag (45°/−45°), and concentric were also investigated along with the previously reported patterns [12]. These raster patterns are commonly used on slicing software such as Cura version 4.8 (Ultimaker Cura, Framingham, MA, USA). Due to the influence of temperature on semi-crystalline polymers and to represent the deposition process in FDM, in this work element activation and heat transfer are considered along with modified crystallisation physics that was initially developed by Levy A [30]. The simulation results were validated by comparing the warpage of the FDM printed parts against the simulation results.

## 2. Materials and Methods

### 2.1. Process Parameters Description

In FDM, the process parameters considered in this study along with their terms are described here as follows:

- Print bed—Also commonly referred to as build plate is used as a base for deposition and cooling of the extruded filament from the nozzle in FDM process. For better adhesion and print quality, it is vital to print on a clean and heated print bed [17].
- Ambient temperature—The room temperature in a FDM chamber while the part is being printed is referred to as ambient temperature. It is also commonly referred to as chamber temperature.
- Extrusion temperature—It is the temperature at which the feedstock is extruded from a FDM nozzle.
- Print speed—Otherwise known as nozzle speed, is the speed at which the filament is deposited on the print bed during the deposition process. Print speed is expressed in mm/s units.
- Nozzle diameter—The diameter of the nozzle used for the extrusion process in FDM is referred to as nozzle diameter.
- Layer thickness—It is the thickness of an individual layer (measured on the Z-axis) of an FDM printed part. This parameter can be influenced by nozzle diameter as layer thickness of a part is always smaller than the respective nozzle diameter used for its printing process [31].
- Infill density—In FDM, the feedstock material is melted and deposited to fabricate a 3D object. In order to achieve the solid shape, the part is filled with melted feedstock which later cools down to form the object. This is commonly referred to as infill and is represented in percentage (%). The infill density can significantly influence the printing time and therefore the mechanical properties of a printed part.
- Raster pattern—In FDM, the printed part is filled in various shapes/patterns. These patterns are often referred to as raster pattern/infill pattern/print pattern etc. Generally, raster pattern of a part can be defined with the aid of a slicing software.
- Roads—Raster pattern is comprised of filaments deposited adjacent to each other forming a layer which is commonly known as roads [25]. Depending on the selected raster pattern, the roads could be shorter and longer. For example, in line (90°/90°) and (0°/90°) the raster pattern is made of long roads entirely. However, in zigzag (45°/45°) and (45°/−45°), the raster pattern initiates with shorter roads followed by long roads and then ending with short roads.

In this study, isotactic polypropylene (PP) provided by 3D Fila (Essex, UK), was selected as the material study as it is one of the commonly used semi-crystalline polymer feedstock in FDM. The study samples were printed and simulated under the following conditions: bed temperature 100 °C, ambient temperature 25 °C (room temperature), print speed of 30 mm/s, a layer thickness of 0.5 mm using a nozzle diameter of 0.8 mm with an infill of 100% in order to investigate the resulting overall warpage from the printed samples. The raster pattern investigated in this study was selected from the slicing software Cura version 4.8 (Ultimaker Cura, Framingham, MA, USA), then printed using the modified Ultimaker 2. The Ultimaker was modified by placing a glass door in the front of FDM along with a glass chamber on the top of the printer in order to maintain a constant ambient temperature while the print is being carried out. These printed samples were later simulated using COMSOL Multiphysics software (Version 5.5, COMSOL, Cambridge, UK). The warpage from the printed samples was experimentally measured using an Absolute arm (8525 model) with a RS6 scanner for higher accuracy (GH Inspection LTD, Cambridge, UK). The various raster patterns that are considered in this study are presented in Table 1.

**Figure 1:**
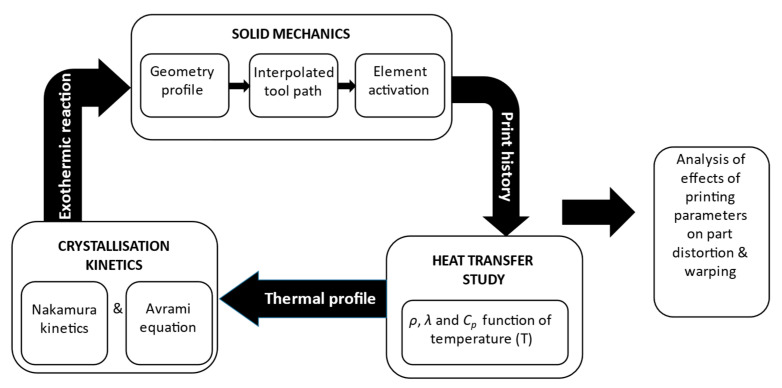
Process plan.

The simulation study presented here is a multi-physics model comprising physics such as solid mechanics, heat transfer, and polymer crystallisation kinetics as shown in Figure 1. The geometry of the model is designed as represented in Figure 1, through solid mechanics. In order to simulate the part distortion resulting from the FDM process accurately, element activation has been incorporated in this study through solid mechanics physics. An in-house built tool path is invoked through the element activation technique thus activating the meshed elements similarly to the FDM deposition process. Among the various element activation techniques available in COMSOL Multiphysics, the models in this study are activated with respect to the material deposition in line with their respective raster pattern. In heat transfer studies, the thermo-mechanical material properties such as specific heat (*C_P_*), density (*ρ*), and thermal conductivity (*λ*) are assigned to the model as functions of temperature (*T*). Since semi-crystalline polymers are highly temperature-dependent, this approach was taken in order to avoid assigning constant values to the thermo-mechanical properties of the polymer. Thus, enabling the thermo-mechanical material properties of the model to vary continuously from the point the material is deposited until it is cooled down. These data are transferred to crystallisation kinetics where the crystallisation phenomenon-based equations such as Nakamura kinetics and Avrami equations are incorporated into the model. Since all the incorporated physics in this simulation model are coupled with respect to temperature (*T*), the simulation model constantly evolves with respect to crystallisation and heat transfer throughout the deposition process.

However, due to the complexity of the simulation model and taking into consideration the computation time, the simulated sample dimensions were restricted to 50 mm × 50 mm × 2 mm (4 layers, each layer with a thickness of 0.5 mm). Along with the incorporated crystallisation kinetics and thermo-mechanical material properties, this simulation study also takes into consideration factors including gravity, temperature transfer and the contact between the print bed and the deposited filament, ambient temperature, and the viscoelasticity of polypropylene (PP).

### 2.2. Physics Interfaces

#### 2.2.1. Solid Mechanics

Initially, after designing, the model is meshed with respect to the size of the roads deposited from the FDM nozzle (0.5 mm in this study). Through solid mechanics, the meshed element of the geometry is activated with respect to its X and Y co-ordinate similar to the FDM deposition process. For this study, in-built tool path programs are written and imported into the simulation. These raster pattern codes contain X and Y co-ordinates of the respective raster patterns along with their deposition time. Thus, coupled with time, each meshed element of the model is activated with respect to the material deposition process. Once all the elements of a layer are completely activated, the element from the subsequent layer activates resembling the nozzle travelling in the Z-axis in FDM process for continuous deposition process.

**Figure 2:**
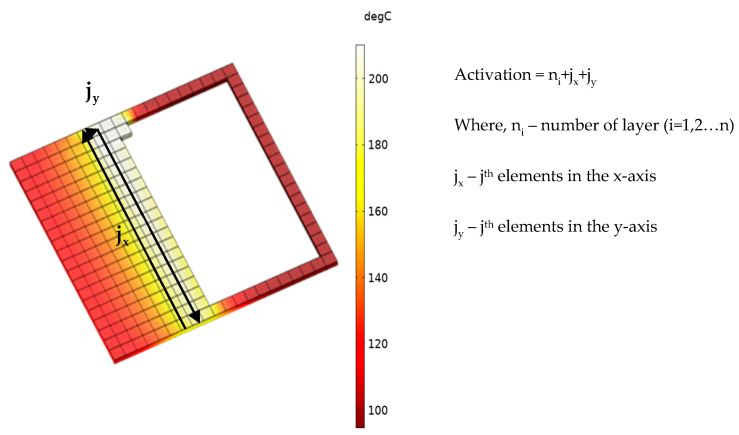
Representation of element activation with respect to the material deposition.

As illustrated in Figure 2, elements in the first layer of the simulated model are activated with respect to the material deposition (X and Y co-ordinates). Since this is a continuous element activation process, the newly deposited elements re-heat the previously deposited elements throughout the process. This temperature transfer effect between the deposited roads and layers has been taken into consideration in this study. In-house tool path programs were written in order to simulate raster patterns such as line (90°/90°), line (0°/90°), zigzag (45°/45°), zigzag (45°/−45°), and concentric. In the developed simulation model, the print bed is represented with a fixed temperature boundary condition under the bottom layer (layer 1) of the models. To simulate the warping behaviour of the FDM printed part, a spring foundation boundary condition was used, as it provides a degree of freedom to the model thereby allowing it to detach from the print bed while being cooled. Spring foundation boundary condition has been successfully incorporated and reported by other simulation studies as well [32,33].

#### 2.2.2. Heat Transfer Study

Since semi-crystalline polymers are highly temperature dependent and crystallisation phenomenon is an exothermic process, the thermo-mechanical properties of PP were coupled with the temperature gradient of the model. Furthermore, factors such as heat transfer and rate of cooling can significantly affect the final mechanical properties of the printed part. Hence in the literature, significant emphasis has always been placed on heat transfer in FDM simulation studies [13]. Therefore, in this study, the incorporated physics (solid mechanics, heat transfer, and crystallisation kinetics) have been coupled with the temperature (*T*) of the model. The general energy balance equation used in this study for heat transfer is presented in Equation (1):

(1) $$\rho C_{p}\frac{\partial T}{\partial t}-\nabla .\left(\lambda \nabla T\right)=Q$$

Here, ρ is the density, $C_{p}$ is the specific heat capacity, λ is the thermal conductivity, and Q is the heat source.

#### 2.2.3. Crystallisation Kinetics

In order to achieve accurate simulation of part distortion of FDM printed semi-crystalline parts, the thermo-mechanical properties (thermal conductivity (λ), specific heat capacity ($C_{p}$) and density (ρ)) of the material of study (PP) were taken into consideration. In the literature, it has been reported that these thermo-mechanical properties can significantly influence the thermal gradient of a polymer [34,35]. Therefore, these material properties (λ, $C_{p}$, ρ) are invoked in the simulation as expressions in terms of the function of temperature (*T*). Using the rule of mixture, the solid and liquid state of these material properties weighted by relative crystallinity is given in Equations (2)–(4):

(2) $$C_{p}\left(\alpha ,T\right)=\alpha C_{psc}\left(T\right)+\left(1-\alpha \right)C_{pa}\left(T\right)$$

(3) $$\lambda \left(\alpha ,T\right)=\alpha \lambda_{sc}\left(T\right)+\lambda_{a}\left(T\right)$$

(4) $$\rho \left(P,\alpha ,T\right)=\alpha \rho_{sc}\left(T\right)+\left(1-\alpha \right)\rho_{a}\left(T\right)$$

In Equations (2)–(4), the subscript *sc* indicates the semi-crystalline regions and *a* represents the amorphous regions of the polymer. The thermo-mechanical expressions of both amorphous and semi-crystalline regions of polypropylene are given in Table 2.

As a result of incorporating these functions, the material properties of the polymer changes in the simulation with respect to the change in temperature experienced while the polymer is undergoing phase change during cooling process.

Taking into consideration non-isothermal crystallisation conditions, Nakamura extended Avrami equation as [37]:

(5) $$\alpha \left(t\right)=1-exp{\left[-\overset{t}{\underset{0}{\int }}K\left(T\right)dt\right]}^{n}$$

Here, t represents time, n indicates Avrami index and K(T) depicts the Nakamura crystallisation kinetics function. Later, Koscher et al. proposed an expression for K(T) for both isothermal and non-isothermal conditions through their experimental study. The K(T) they proposed is written as [38]:

(6) $$K\left(T\right)={\left(\frac{4}{3}\pi N_{0}\left(T\right)\right)}^{\frac{1}{3}}G_{0}\times \exp\left(-\frac{U^{*}}{R\left(T-T_{\infty }\right)}\right)exp\left(-\frac{K_{g}}{T\left(T_{f}-T\right)}\right)\;$$

In Equation (6), $N_{0}=exp\left(0.156\times \left(T_{f}-T\right)+15.1\right)$, where, $G_{0}$ = 2.83 × $10^{2}$, $K_{g}$ = 5.5 × $10^{5}$ ($K^{2}$), $U^{*}$ = 6284 J/mol. *K*, *R* is the gas constant, $T_{f}$ = 210 °C, $T_{\infty }$ = $T_{g}$ − 30 °C, ΔH = 90 ×$\;10^{3}$ J/kg, and n = 3.

Since the invoked Nakamura equation incorporates both time (*t*) and temperature (*T*), they both are taken into consideration as the driving factor for the simulation [30,39,40]. Owing to the incorporation of the modified crystallisation physics, the simulated model can predict the part distortion occurring in a semi-crystalline polymer printed using FDM. An in-depth explanation of these incorporated physics, boundary conditions, and the expressions invoked in them has been discussed in detail in our previous works [12,14].

## 3. Results and Discussion

**Figure 3:**
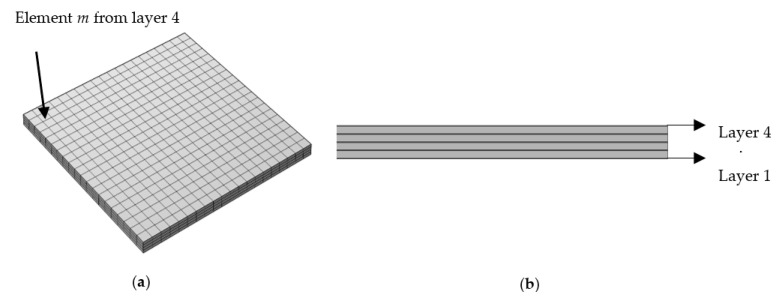
(**a**) Iso-metric view and location of element *m* in the printed part (**b**) Side view of the printed part representing the layer sequence.

**Figure 4:**
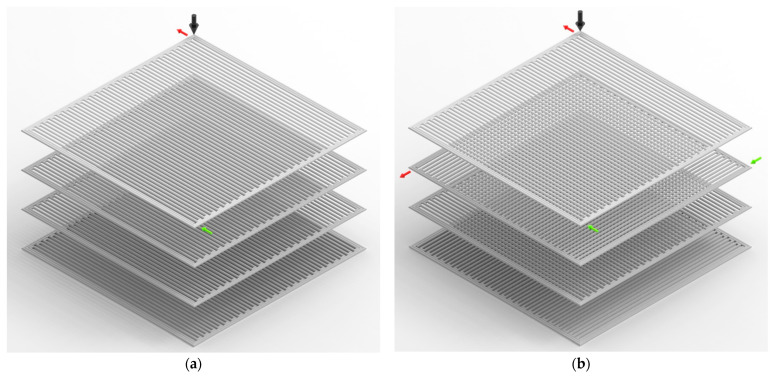

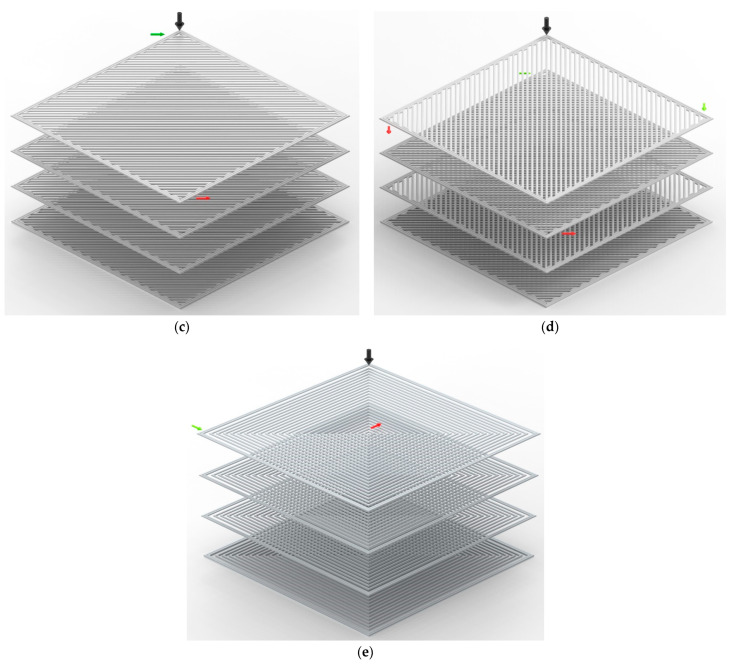
FDM raster patterns considered in this study along with their initial and final deposition position. (**a**) Sample a-Rs (90°/90°), (**b**) Sample b-L (0°/90°), (**c**) Sample c-Z (45°/45°), (**d**) Sample d-Z (45°/−45°), and (**e**) Sample e-Con. The raster patterns presented here are only a representation of the actual printed FDM patterns. Here, the black arrow represents the location of element *m*, the green arrow denotes the initial direction of the respective print while the red arrow shows the direction the print terminates for that respective layer.

In the simulated models, in order to analyse the impact of various FDM raster patterns, a specific element was selected from the top layer (layer 4). Throughout the study, this element will be referred to as element *m*. Element *m* has the co-ordinates 7.8, 2.1, and 1.5 mm (X, Y, Z co-ordinates). An element from the top layer was selected because it is being deposited on the previously deposited layer (third layer) and the thermal gradient from the heated roads/layers surrounding the element can be studied while it is cooling down. The location of element *m* in layer 4 along with the sequence of the layers of the simulated/printed model in iso metric view is presented in Figure 3a. In Figure 4, the raster patterns that are considered in this study along with the layer-by-layer representation including the deposition origination and termination of the third layer and the fourth layer are presented. To provide a visual representation of the alternate patterns in sample b-L (0°/90°), and d-Z (45°/−45°), and their effect on element m print directions of third and fourth layers are displayed in Figure 4.

In this study, the temperature transfer between the roads, accumulated residual stresses, and warpage results obtained from samples b-L (0°/90°), c-Z (45°/45°), d-Z (45°/−45°), and e-Con are compared with the reference sample (sample a-Rs (90°/90°)).

### 3.1. Changes in Temperature with Printing Time

**Figure 5:**
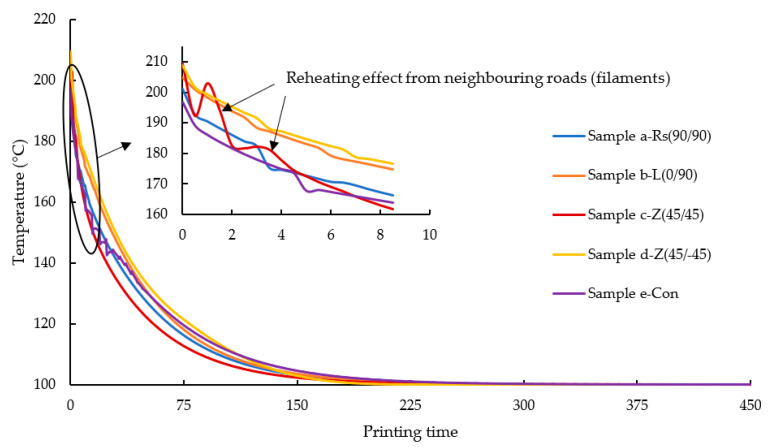
Comparison of thermal history of element *m* (selected from the top layer) from the models simulated under various raster pattern. An inset plot is attached to magnify the reheating effect received by element *m* from its neighbouring roads.

The main aim of this section is to understand the impact of FDM raster patterns on the thermal gradient of the printed/simulated sample. Figure 5 represents the temperature gradient of element *m* from deposition at 210 °C onto the previous layer (layer 3) until it reaches the bed temperature (100 °C). The simulation results of all the samples a-Rs (90°/90°), b-L (0°/90°), c-Z (45°/45°), d-Z (45°/−45°), and e-Con are rescaled and overlapped in order to investigate the impact of various raster patterns on the thermal history of element *m*. Although FDM is a continuous process, in order to investigate the temperature transfer around element *m* (since the time of deposition of element *m*), in this study, the time scale has been rescaled to start from t=0 sec and end when the temperature of element *m* reaches the bed temperature (100 °C). It should be noted that in reality, these raster patterns have different printing times. For comparison purposes, the x-axis (dimensionless time) of the graphs starts from 0 s.

In Figure 5, it can be seen that element *m* cools down rapidly from 210 °C (the deposition temperature) in all samples due to the heat loss caused by the lower ambient/surrounding temperature.

**Figure 6:**
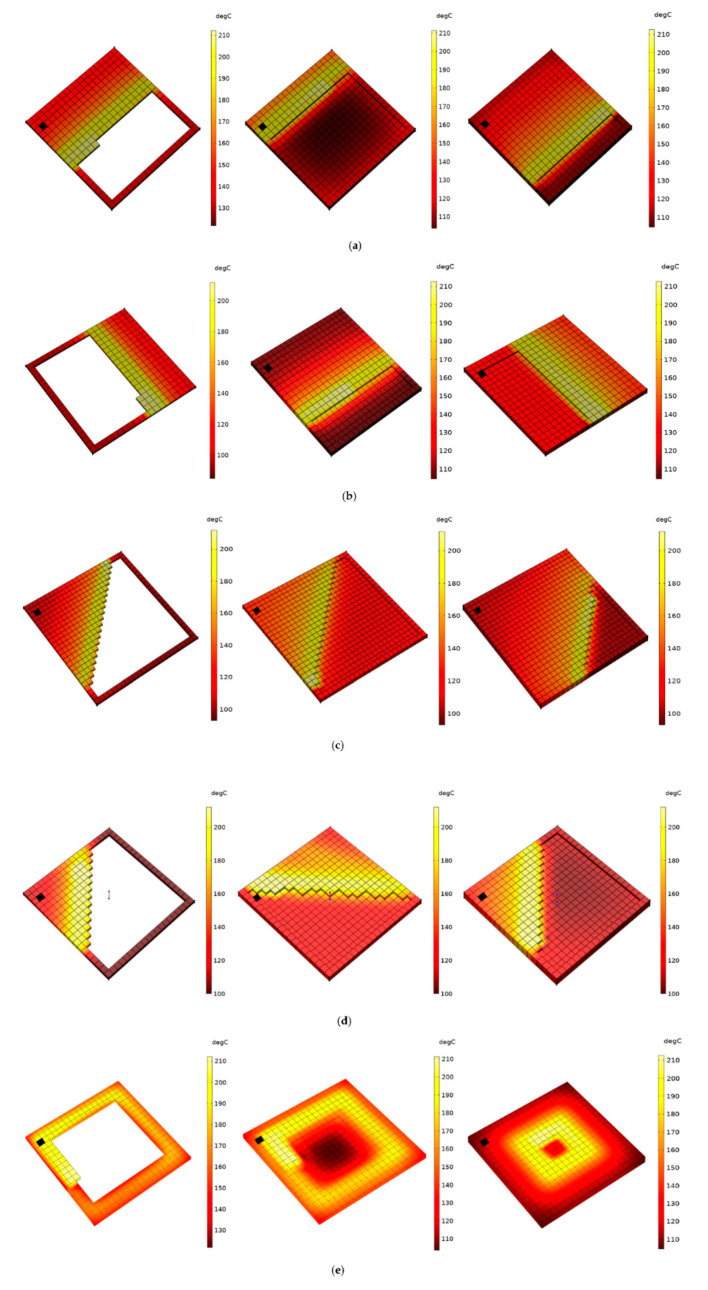
Representation of sequential element activation of various raster patterns considered in this study with respect to their material deposition. (**a**) sample a-Rs (90°/90°), (**b**) Sample b-L (0°/90°), (**c**) sample c-Z (45°/45°), (**d**) sample d-Z (45°/−45°) and (**e**) sample e-Con. A black marker is placed in all the samples to illustrate the location of element *m* in order to understand the reheating effect imposed on it by the adjacent road deposition.

From Figure 5, it is evident that sample c-Z (45°/45°) cools more rapidly compared to Z (45°/−45°). In the FDM process, when the roads are deposited continuously next to each other, the heat from the newly deposited road would reheat the previously deposited roads. This phenomenon can be observed in the inset plot of Figure 5, where the thermal history of element *m* from sample c-Z (45°/45°), increases and decreases, while in the other samples, the reheating effect is much less visible and the temperature decreases almost monotonically. As can be seen in Figure 4c and Figure 6c, since the roads on layer 4 in sample c-Z (45°/45°) are much shorter in comparison to the other samples in the region of element *m*, here, element *m* gets reheated multiple times by the adjacent neighbouring roads. To a certain extent, this phenomenon can also be seen in sample e-Con where there are minor peaks observed below 160 °C. However, despite the reheating effect from neighbouring roads, sample c-Z (45°/45°) displays a faster cooling rate than all the other samples. This is because, as the newly deposited roads move further away from element *m* and become longer roads, the reheating effect received by element *m* is greatly reduced leading to faster cooling. This effect is also seen in samples a-Rs (90°/90°), and e-Con, where the distance between the newly deposited roads and element *m* increases. While in sample b-L (90°/90°) and d-Z (45°/−45°), due to their alternating pattern (as seen in Figure 4b,d), element *m* lies inside the vicinity of the newly deposited roads thereby resulting in gradual cooling compared to the other samples.

During the initial cooling period, sample e-Con resembles sample a-Rs (90°/90°) and c-Z (45°/45°), however, later, minor peaks can be observed from the inset plot in Figure 5. In sample e-Con, due to the concentric raster pattern, the outer roads take a longer time to be deposited (as seen in Figure 4). Relative to sample c-Z (45°/45°), the adjacent neighbouring road that is in close proximity to element *m* in sample e-Con is deposited after a longer period of time, due to which minor reheating peaks can be seen when element *m* is cooling down below 160 °C. Among all the samples, it can be observed that only element *m* from sample e-Con experiences reheat after being cooled below 160 °C. Owing to the long roads of this specific raster pattern, element *m* gets reheated periodically from the nearby newly deposited adjacent roads. As a result, the cooling rate of element *m* from sample e-Con is higher than in the other samples.

Contrary to samples a-Rs (90°/90°) and c-Z (45°/45°), samples b-L (0°/90°) and d-Z (45°/−45°) appear to have a slower cooling rate. This is mainly because of the alternating raster patterns as shown in Figure 4b,d; element *m* is deposited over the very recently deposited roads from the third layer. In the other samples (a-Rs (90°/90°), c-Z (45°/45°), and e-Con), when element *m* is deposited, the region below element *m* from the third layer has cooled down significantly, while in samples b-L (0°/90°) and d-Z (45°/−45°), the roads below element *m* still retain a high temperature while element *m* is being deposited, thus resulting in a much slower cooling rate.

Despite the alternative raster patterns, the trend of the cooling curve of element *m* from sample b-L (0°/90°) closely resembles that of sample a-Rs (90°/90°). This is because the raster angle of both the samples’ final layer (layer 4) is 90° (as seen in Figure 4). Therefore, there is not a significant change noticed in the trend other than the slow cooling rate of element *m*.

### 3.2. Changes in Residual Stress with Printing Time

**Figure 7:**
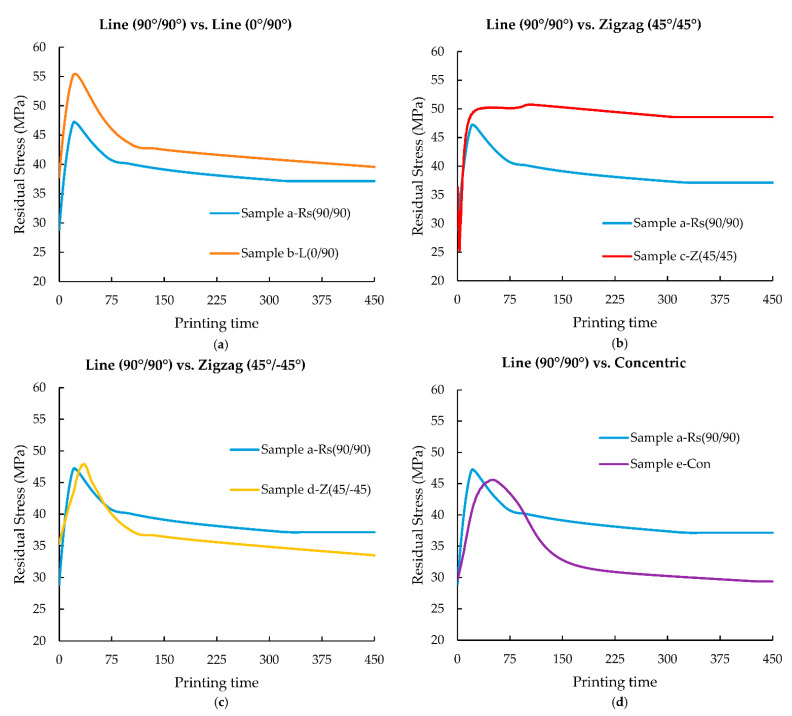
Comparison of residual stress results from (**a**) sample b-L (0°/90°), (**b**) c-Z (45°/45°), (**c**) d-Z (45°/−45°), (**d**) e-Con plotted against the printing time (dimensionless) and compared with sample Rs (90°/90°) (reference sample).

As discussed in Section 3.1, parts printed via the FDM process undergo anisotropic cooling leading to the accumulation of internal thermal residual stresses. Cooling during the FDM process can be affected by various printing parameters such as raster pattern, which can, in turn, lead to different amounts of residual stress in parts of the same design. As seen in Figure 6 (Section 3.1), depending on the raster pattern, the reheat supplied to element *m* is significantly affected by the newly deposited adjacent roads around it. Furthermore, the cooling pattern of the complete printed part also changes with respect to the selected raster pattern. For instance, in sample e-Con (concentric pattern), the overall cooling of the part begins from outward to inward as the deposition process progresses. However, in terms of sample a-Rs (90°/90°), since the printing pattern proceeds from one side of the part to the other side, the part cools along with the deposition of the material (from one side to another), thus leading to a non-homogenous cooling compared to sample e-Con. This is also applicable to samples b-L (0°/90°), c-Z (45°/45°), and d-Z (45°/−45°). Depending on the cooling pattern of a printed part, a considerable difference can occur in the accumulation of thermal residual stresses. Figure 7a–d depict the evolution of residual stresses in element *m* in samples a-Rs (90°/90°), b-L (0°/90°), c-Z (45°/45°), d-Z (45°/−45°), and e-Con. Here, the residual stresses from samples b-L (0°/90°), c-Z (45°/45°), d-Z (45°/−45°), and e-Con are compared against the stress results from sample a-Rs (90°/90°). Similar to the previous section, the residual stresses results are rescaled with respect to printing time so that residual stress profiles can be more easily compared against the reference sample (sample a-Rs (90°/90°)) results.

In all the graphs in Figure 7, it is evident that the residual stress value initiates at a high value during the initial deposition stages of element *m*, followed by a predominant peak representing a significant accumulation of residual stress, which is then followed by a gradual drop in the accumulated stress as element *m* is allowed to cool. This increase in residual stress during the initial deposition of element *m* can be explained by the stress development in the deposited material caused due to the significant temperature difference between element *m* and the third layer. Here, PP is deposited at around 210 °C, while the bed temperature was maintained at 100 °C and the ambient temperature was maintained at 25 °C. Even though element *m* was deposited on top of layer 3, on deposition the temperature of the material around element *m* is much lower than the deposition temperature of element *m*. This low-temperature gradient gives rise to a significant temperature difference resulting in a non-homogeneous cooling leading to thermal residual stresses trapped inside the roads/layers [41]. Additionally, crystallisation in semi-crystalline polymer parts results in volumetric change (shrinkage) which further contributes to the increase in the accumulated residual stresses in the samples presented in Figure 7 [13]. These internally developed residual stresses can be released through relaxation of the polymer chain over time and through reheating [42]. From the graphs in Figure 7, it can be seen that as element *m* cools down, the accumulated stress gradually drops.

Relative to the other samples, it can be noted from Figure 7b that the residual stress trend of element *m* from sample c-Z (45°/45°) displays a sudden drop during the initial stage of deposition followed by a significant increase. As seen from Figure 4c and Figure 6c, compared to the other samples, the initial roads of sample c-Z (45°/45°) are much shorter and therefore it takes a short amount of time for the neighbouring roads deposition to take place, which leads to more frequent reheating of element *m*. Even though due to the initial temperature difference between the deposited element *m* and the surrounding temperature, the initial residual stress of element *m* originates at 36 MPa, due to these frequent and constant reheats, a considerable drop in the stress curve is noted. However, as already explained in Section 3.1, the cooling pattern of a FDM printed part is evidently influenced by its raster pattern; since element *m* is located in the corner of sample c-Z (45°/45°), as the distance between the newly deposited roads and element *m* increases, element *m* cools faster trapping the thermal stresses accumulated [15,19] when compared to the other samples. Sample c-Z (45°/45°) shows an increase of 31% in residual stresses exhibiting the highest stresses among all the samples.

On comparing samples a-Rs (90°/90°) and b-L (0°/90°), the stress profiles appear to be similar; however, sample b-L (0°/90°) shows an increase of 6.5% in residual stress relative to sample a-Rs. As already mentioned in Section 3.1, the direction of cooling also influences the residual stresses in FDM printed parts owing to their respectively selected raster patterns [19]. In sample a-Rs (90°/90°), since the layers are deposited unidirectionally, the roads are cooled from one side to another. However, in sample b-L (0°/90°), the layers are deposited in an alternating pattern. In sample b-L (0°/90°), layer 3 (the layer below element *m*) was deposited right to left (with 0° raster angle), and when element *m* was deposited, the raster pattern begins from the bottom of sample b-L (0°/90°) and terminates at the top (as seen from Figure 4b). In other words, the area under element *m* was printed at the end of layer 3, so the region underneath it has significantly cooled down. Therefore, when element *m* is being deposited the surrounding temperature is much lower than its deposition temperature. This leads to a significant increase in the accumulation of residual stresses between the layers [41], which is clearly seen here. Due to this increased accumulation of thermal stress, the initial stress value of element *m* is at a much higher value and the peak found in sample b-L (0°/90°) is observed to be wider than the peak found in sample a-Rs (90°/90°) (reference sample). Mainly because in sample b-L (0°/90°), as a larger amount of thermal stresses are being trapped, it requires a longer time to relax as can be seen from Figure 7.

The sample d-Z (45°/−45°) residual stress curve resembles the stress curves from samples a-Rs (90°/90°), b-L (0°/90°), and e-Con. This is because in all of these samples, element *m* is present in a long road and there is not much difference in the stress curve noted here. Thus, a decrease of 9.8% in residual stress is observed here. However, the stress peak found in sample e-Con is evidently wider than the peak noted in the other samples. This is because of the long and continuous roads being deposited in sample e-Con compared to the other samples. Similar to the reheat received from the adjacent neighbouring roads, element *m* can also continuously experience heat from the road that is being deposited along with it (as seen in Figure 4e). However, the roads in sample e-Con are continuous and long in comparison to the other samples in this study. Therefore, due to this phenomenon, the accumulated stress in element *m* is released slowly depicting a wider peak displaying the lowest residual stress. Due to these long continuous roads, the residual stress in sample e-Con decreases by 21% relative to the reference sample. Pandzic et al. and Nickel et al. studied the mechanical properties of various FDM raster patterns. In their study, they reported that the Concentric raster pattern displayed the highest ultimate tensile and yield strength. Their study agrees with the simulated result of sample e-Con here, where the lowest residual stresses were noted, and thus superior mechanical properties would be expected compared to the other FDM raster patterns [27,28].

### 3.3. Evolution of Warpage throughout the Printing Process

**Figure 8:**
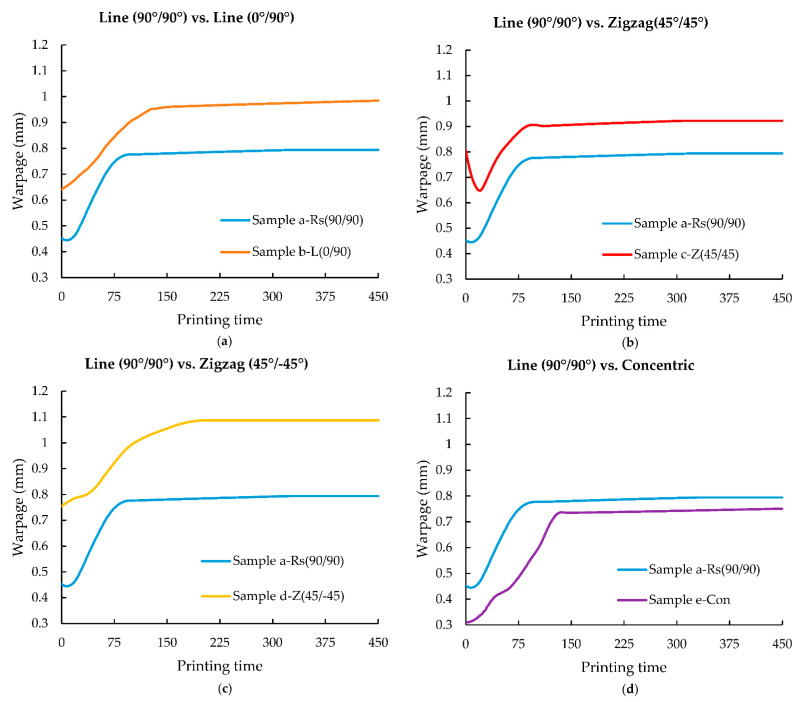
Comparison of warpage results from (**a**) sample b-L (0°/90°), (**b**) c-Z (45°/45°), (**c**) d-Z (45°/−45°), (**d**) e-Con plotted against the printing time (dimensionless) and compared with sample Rs (90°/90°) (reference sample).

As a result of the anisotropic contraction caused by the accumulation of thermal residual stresses and volumetric contraction induced by polymer crystallisation, the FDM printed parts experience warpage [43,44]. Translation of the residual stresses observed in Figure 7 (Section 3.2) into warpage can be seen in Figure 8. Warpage in 3D printing can be defined as a change in the dimension/geometry of a material when exposed to heated or cold environmental conditions. In this study, warpage is reported with respect to the z direction of the printed/simulated models. Similar to residual stresses, the warpage trend of element *m* from every sample is also plotted and compared against the reference sample’s (sample a-Rs (90°/90°)) warpage in Figure 8 against their printing time.

From Figure 8, it is evident that the warpage of element *m* in all samples increases steadily from the time of deposition. This increase in warpage reaches a plateau as the sample cools down. Additionally, during the initial deposition stage, a slight drop in warpage can be seen in Figure 8. While this drop in warpage is not observed in all the samples, this is pronounced in samples a-Rs (90°/90°) and c-Z (45°/45°) (Figure 8b). During the initial deposition, due to the temperature received from the neighbouring roads from their close proximity, a drop in the warpage is noted. However once element *m* begins to cool down, a steady increase in warpage occurs.

In Figure 8a, the increase in warpage is more gradual in sample b-L (0°/90°). This effect was also seen in the residual stress trend of sample b-L (0°/90°) compared to sample a-Rs (90°/90°). This effect was also seen in the residual stress trend of sample b-L (0°/90°). As previously explained in Section 3.2., the region below element *m* was deposited at the end of layer 3 deposition, and element *m* was printed at the end of layer 4 deposition. Due to this, the region below element *m* has cooled causing significant temperature difference resulting in higher residual stress and warpage as seen in Figure 7a and Figure 8a. This explanation can also be used to corroborate element *m* from sample b-L (0°/90°) exhibiting high initial residual stress and warpage values compared to sample a-Rs (90°/90°). Sample b-L (0°/90°) shows a significant increase of 24% in warpage.

In Figure 8b, a steep drop in warpage is noted in sample c-Z (45°/45°) in the initial printing stage when compared with sample a-Rs (90°/90°) (reference sample). This is because, in sample c-Z (45°/45°) when element *m* is being deposited, shorter adjacent roads are deposited sequentially within a short period of time, which results in lowering the temperature gradient around element *m*, due to which the warpage continues to decrease. This phenomenon can also be seen in the residual stress trend of element *m* from sample c-Z (45°/45°). Since element *m* in sample a-Rs (90°/90°) is deposited at the end of layer 4, no significant heat is received from the adjacent roads and therefore no notable decrease in warpage is seen. Similar to sample b-L (0°/90°) and c-Z (45°/45°), the initial warpage value of element *m* in sample d-Z (45°/−45°) also begins from a higher value in comparison to element *m* in sample a-Rs (90°/90°) (reference sample). However, there is no warpage drop observed in sample d-Z (45°/−45°), furthermore, the increase in warpage of element *m* from sample d-Z (45°/−45°) resembles closely the warpage trend from sample b-L (0°/90°). Due to the alternate raster pattern in both these samples, when element *m* is being deposited there is a considerable difference in the temperature. In sample d-Z (45°/−45°), the region below element *m* (layer 3) is deposited during the initial deposition stage of layer 3 (similar to sample c-Z (45°/45°)). However, due to the alternate raster pattern in sample d-Z (45°/−45°), element *m* is deposited in the middle of the layer deposition of the whole layer 4. There is a significant time difference between the region below element *m* being deposited and element *m* being deposited in these cases, thus, leading to the development of internal residual stresses resulting in warpage. Due to the large accumulation of residual stresses, the stress peaks (Figure 7b,c) and warpage in sample b-L (0°/90°) and d-Z (45°/−45°) (Figure 8b,c), appear to be much wider than the stress peak and warpage trend in sample a-Rs (90°/90°) (reference sample).

Sample e-Con displays the lowest residual stress and warpage of all samples. As can be observed in Figure 7d, the residual stress value of sample e-Con initiates at a considerably lower value than the other samples. This is also translated into warpage, as can be seen in Figure 8d. Even though sample e-Con exhibited a decrease of 21% residual stress when compared with sample a- Rs (90°/90°), in terms of warpage only a 5.5% decrease in warpage is noted. Although the long roads in sample e-Con lead to relaxation and release of the thermal residual stresses trapped in element *m* (as seen in Figure 7d), the resultant warpage here could be related to polymer crystallisation. The wider peak of residual stress can be seen in Figure 7d, as the warpage in element *m* gradually and steadily increases in comparison to sample a-Rs (90°/90°).

### 3.4. Comparison between the Final Residual Stress and the Overall Warpage

**Figure 9:**
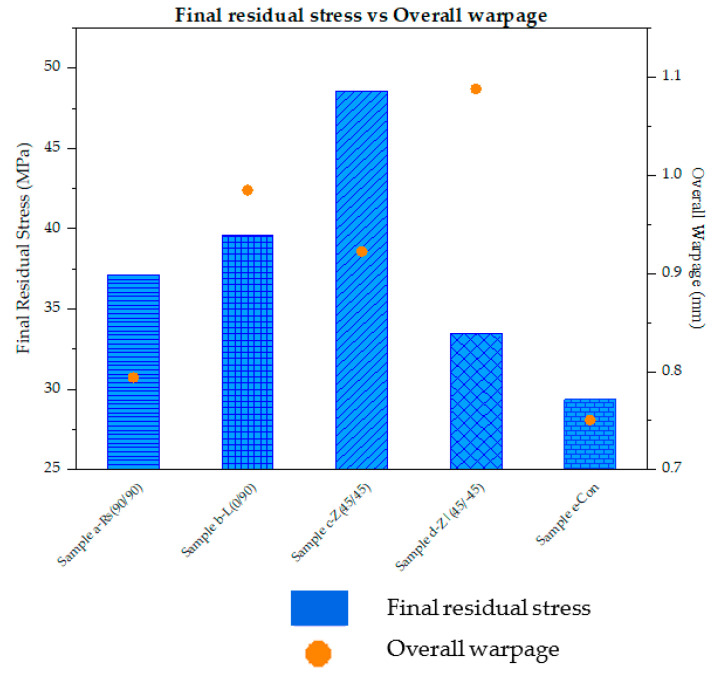
Comparison of final residual stress and overall warpage results from element *m* of sample a-Rs (90°/90°), b-L (0°/90°), c-Z (45°/45°), d-Z (45°/−45°), and e-Con.

The final residual stress values of element *m* for the considered raster patterns are plotted in Figure 9 and compared with their respective overall warpage results. The patterns in each bar (representing the final residual stress value) indicate their respective raster pattern.

From Figure 9, it can be seen that degree of warpage overall follows the thermal residual stresses developed in the samples. When compared with sample a-Rs (90°/90°), sample b-L (0°/90°) shows a 6.6% increase in residual stress along with a 24% increase in warpage, while sample c-Z (45°/45°) exhibits a significant increase of 31% and 16.2% in residual stress and warpage, respectively. In both samples (sample b-L (0°/90°) and c-Z (45°/45°)), with an increase in residual stress, an increase in warpage is also observed. As mentioned in Section 3.1. and Section 3.2., due to the alternating patterns, the residual stress in sample b-L (0°/90°) increases leading to an increase in warpage, while in sample c-Z (45°/45°), since the deposition begins with element *m* and proceeds to the other end of the sample, the cooling pattern follows the same path. This leads to trapping the internally developed residual stresses. In sample d-Z (45°/−45°), due to the long raster of element *m*, the accumulated residual stress is relaxed and released leading to a decrease of 9.8% stress, which is noticed here. However, in terms of warpage, compared to sample a-Rs (90°/90°), sample d-Z (45°/−45°) shows an increase of 37% in warpage. From Figure 9, it is evident that sample e-Con displays the lowest accumulated residual stress and warpage. Sample e-Con depicts a 21% decrease in residual stresses and a 5.5% decrease in warpage. Similar to sample d-Z (45°/−45°), the decrease in residual stress in sample e-Con can be attributed to the longer road of element *m*, where it is allowed to cool down. Here in both samples, the adjacent neighbouring roads to element *m* are deposited some time after element *m* is being printed. In sample e-Con, this neighboring road is deposited much later than in sample d-Z (45°/−45°). Due to the long roads, even though the accumulated residual stress in both sample d-Z (45°/−45°) and sample e-Con decrease, the warpage results do not follow their stress trend. In sample d-Z (45°/−45°), element *m* is deposited in the middle of the layer deposition of layer 4. Furthermore, in this sample, due to the 45°/−45° raster pattern, element *m* only receives heat from the immediate neighbouring road. However, in sample e-Con, element *m* is being deposited during the initial deposition stage of layer 4. Therefore, due to this and the raster pattern, element *m* in sample e-Con constantly receives considerable continuous heat from the neighbouring roads. This leads to a decrease in both thermal residual stresses and resulting warpage.

## 4. Experimental Validation

The experimentally printed samples were 3D scanned using an absolute arm (8525 model) with a RS6 scanner for increased accuracy. For this, a cartesian co-ordinate was created at co-ordinates 0, 0, 0 (X, Y, and Z axis); this axis was considered as the nominal axis. Another axis was created at the co-ordinates corresponding to element *m* (7.8, 2.1, and 1.5 mm). For each sample, the deviation of their respective element *m* was measured from the nominal axis constructed at the location of element *m*.

From Table 3, it is evident that the simulated warpage results obtained from the developed model under predicts the experimental warpage values. However, the predicted warpage results are still in good agreement with the experimentally measured warpage values.

## 5. Conclusions

In this study, the effect of various commonly used FDM raster patterns on part distortion in polypropylene (a semi-crystalline polymer) was investigated. In this study, a sample with the raster pattern line (90°/90°) was used as a reference sample to assess the effect of the other raster patterns in the study.

From the results, it can be seen that among all the considered raster patterns, the sample with the concentric raster pattern displayed the least warpage (5.5% decrease) along with the lowest residual stresses (21% decrease). The highest residual stress (with an increase of 31%) along with a 16.2% increase in warpage was recorded for the sample with a zigzag (45°/45°) raster pattern while the sample with a zigzag (45°/−45°) raster pattern showed the highest warpage (37% increase in warpage) along with a decrease of 9.8% in residual stresses. The sample with line (0°/90°) showed a 6.6% increase in warpage along with a 24% increase in warpage. From the results reported from this study and from the literature, in general, it can be concluded that a concentric pattern would be ideal for models resulting in low part distortion and higher mechanical properties. However, when print parameters such as raster width, and printing orientation are changed, even raster patterns such as line and zigzag seemed to have improved mechanical properties. Although it cannot be ruled out that the printing parameters in FDM are beneficial for one raster pattern, the concentric raster pattern has shown to have superior mechanical and structural properties compared to all the other patterns when printed with similar printing conditions.

It can be concluded that the heat transfer between the roads and the deposition time between the layers is crucial to obtaining parts with low part distortion. This phenomenon can be very well seen in the results presented in this study. Hence, it is concluded that the selection of raster pattern has a considerable effect on the part integrity of a FDM printed semi-crystalline polymer.

## 6. Future Work

In this study, the results were obtained for printing orientation and a 100% infill. In future work, raster patterns along with various printing orientations and varying infill percentages will be investigated.

## Figures and Tables

**Table 1:** Raster patterns considered for the simulation study.

Simulated/Printed Samples	Raster Pattern
Sample a-Rs (90°/90°)	Line (90°/90°)
Sample b-L (0°/90°)	Line (0°/90°)
Sample c-Z (45°/45°)	Zigzag (45°/45°)
Sample d-Z (45°/−45°)	Zigzag (45°/−45°)
Sample e-Con	Concentric

**Table 2:** Material properties of PP [36]. Reprinted with permission from [36]. Copyright 2005 Le Go.ff R.

Thermal Property for Amorphous (a) and Semi-Crystalline (sc) States	Numerical Functions
*C_pa_* (*α*, *T*)	3.1 *T* + 2124
*λ_a_* (*α*, *T*)	−6.25 × 10^−5^*T* + 0.189
*ρ_a_* (*α*, *T*)	1/(1.138 + 6.773 × 10^−4^*T*)
*C_psc_* (*α*, *T*)	10.68*T* + 1451
*λ_sc_* (*α*, *T*)	−4.96 × 10^−4^*T* + 0.31
*ρ_a_* (*α*, *T*)	1/(1.077 + 4.225 × 10^−4^*T*)

**Table 3:** Comparison of warpage values from element *m* of various raster pattern samples that were simulated and experimental.

Samples	Predicted Warpage (FEA) (mm)	Measured Warpage (Experimental) (mm)
Sample a-Rs (90°/90°)	0.794	0.8
Sample b-L (0°/90°)	0.98	1.1
Sample c-Z (45°/45°)	0.92	1.14
Sample d-Z (45°/−45°)	1.09	1.25
Sample e-Con	0.75	0.83

## Data Availability

The data presented in this study are available on request from the corresponding author.
